# Extracellular Vesicles in Pregnancy: From Maternal–Fetal Communication to Environmental Perturbation

**DOI:** 10.3390/ijms272110022

**Published:** 2026-11-09

**Authors:** Federica Affranchi, Noemi Aloi, Ilaria Cosentini, Giulia Smeraldi, Paolo Colombo, Gaspare Drago, Silvia Ruggieri, Valeria Longo

**Affiliations:** 1 Institute for Biomedical Research and Innovation, National Research Council of Italy, 90146 Palermo, Italy; federica.affranchi@irib.cnr.it (F.A.); noemi.aloi@irib.cnr.it (N.A.); ilaria.cosentini@irib.cnr.it (I.C.); giulia.smeraldi@irib.cnr.it (G.S.); paolo.colombo@irib.cnr.it (P.C.); gaspare.drago@irib.cnr.it (G.D.); 2 Department of Biological, Chemical and Pharmaceutical Sciences and Technologies (STEBICEF), University of Palermo, 90128 Palermo, Italy; 3 Scuola Universitaria Superiore IUSS Pavia, Piazza Vittoria 15, 27100 Pavia, Italy

**Keywords:** pregnancy, environmental pollutants, exposome, extracellular vesicles, biomarkers

## Abstract

Pregnancy is a uniquely sensitive moment during which environmental exposures may have profound and long-lasting consequences for maternal health, placental function, and fetal development. A successful pregnancy depends on continuous molecular communication among maternal, placental, and fetal compartments. The disruption of these processes by environmental stressors may increase the risk of pregnancy complications, impaired neurodevelopment, and susceptibility to non-communicable diseases later in life. Pregnant women are exposed to dynamic mixtures of chemical, physical, occupational, behavioral, and social stressors from the air, diet, drinking water, consumer products, and residential or workplace environments. The pregnancy exposome framework integrates these external exposures with internal biological responses, including oxidative stress, inflammation, metabolic dysregulation, epigenetic alterations, and changes in circulating biomolecules. However, the mechanisms through which exposure-related signals are transmitted across the maternal–placental–fetal unit remain incompletely understood. Extracellular vesicles may be a key component of this communication network as they transfer protected molecular cargoes, including proteins, lipids, metabolites, and regulatory nucleic acids. Understanding how environmental exposures modify extracellular vesicle biogenesis, composition, and function may reveal mechanisms linking the pregnancy exposome to placental dysfunction, adverse pregnancy outcomes, fetal programming, and early biomarkers of biological effects. This review examines the role of extracellular vesicles in physiological maternal–fetal communication and critically summarizes current human and experimental evidence on how environmental exposures may affect EV characteristics and function during pregnancy, with particular attention to their potential involvement in placental dysfunction and adverse pregnancy outcomes.

## 1. Introduction

Pregnancy is one of the most sensitive periods of a woman’s life course, characterized by profound cardiovascular, metabolic, endocrine, and immunological adaptations that support maternal homeostasis and fetal growth. Throughout gestation, the placenta and developing fetus follow tightly regulated maturation programs that require a precise coordination of endocrine, vascular, metabolic, and immunological processes. This finely balanced physiological state also makes pregnant women particularly vulnerable to environmental perturbations.

During pregnancy, women can be simultaneously exposed to complex mixtures of environmental contaminants from ambient air, drinking water, diet, consumer products, and occupational and residential environments. Environmental pollution has emerged as a major global public health concern, with particularly important implications for pregnant women and their offspring. According to the World Health Organization, approximately 99% of the global population is exposed to air pollution levels exceeding recommended guideline values [1]. However, air pollution is only one component of the broader environmental exposome, which encompasses the complex mixture of chemical, physical, occupational, behavioral, and social stressors encountered throughout life [2]. The scale of this complexity can be captured by the pregnancy exposome framework.

Originally proposed by Christopher Wild, the exposome encompasses the totality of environmental exposures experienced throughout life, together with their corresponding biological responses. It integrates external determinants—including chemical, physical, occupational, and social factors—with internal biological processes such as oxidative stress, inflammation, metabolic dysregulation, altered gene expression, and epigenetic modifications [3]. Applied to pregnancy, this framework captures how maternal exposures interact with gestational physiology to shape both immediate outcomes and longer-term offspring health trajectories [4]. During pregnancy, environmental stressors can disrupt physiological homeostasis and interfere with the tightly regulated maternal–fetal communication network that coordinates placental function, maternal adaptation, and fetal development.

Consistent with this concept, epidemiological studies have linked prenatal exposure to environmental pollutants with an increased risk of pre-eclampsia, gestational diabetes mellitus, fetal growth restriction, preterm birth, and impaired fetal neurodevelopment [5,6,7]. Moreover, environmental insults occurring during critical windows of fetal development may induce persistent biological alterations that increase susceptibility to non-communicable diseases (NCDs) and neurodevelopmental disorders later in life [8,9]. Despite these well-established epidemiological associations, the molecular mechanisms linking environmental exposures to placental dysfunction and altered developmental trajectories remain incompletely understood.

Increasing evidence suggests that environmental stressors leave measurable molecular signatures, including changes in DNA methylation, microRNA (miRNA) expression, protein profiles, metabolites, and circulating extracellular biomolecules, that may represent intermediate molecular responses linking exposure to placental dysfunction, pregnancy complications, impaired fetal development, and long-term disease [10,11,12].

Among the molecular mediators of this communication, extracellular vesicles (EVs) have emerged as key regulators of maternal–fetal crosstalk [13,14,15]. Released by virtually all maternal and placental cell types, EVs transport proteins, lipids, metabolites, messenger RNAs, and non-coding RNAs that contribute to immune tolerance, angiogenesis, vascular remodeling, metabolic adaptation, and fetal development [16]. Because their molecular cargo reflects the physiological state of their cells of origin, EVs may provide a dynamic molecular readout of environmental perturbations while also participating in intercellular communication. Indeed, increasing evidence indicates that exposure to environmental pollutants can alter EVs biogenesis, release, and molecular cargo, including EV-associated proteins and regulatory RNAs, in maternal, placental, and experimental models [13,17]. These exposure-related changes may reflect cellular stress responses and could influence signaling between maternal, placental, and fetal compartments. However, altered EV abundance or cargo alone does not establish that EVs mediate pollutant-induced placental dysfunction or fetal programming. Functional studies demonstrating EV uptake, cargo transfer, or EV-dependent responses in recipient cells remain comparatively limited, and direct causal evidence is restricted to specific experimental models [17]. The present review focuses on the role of EVs in mediating communication across the maternal–placental–fetal unit and in transmitting biological signals associated with environmental exposures. We hypothesized that extracellular vesicles may represent both indicators of environmental stress and potential mediators of altered maternal–placental–fetal communication during pregnancy, although the strength of evidence supporting these roles may differ between human observational studies and experimental models. To identify the most relevant evidence supporting this framework, a structured literature search was conducted using PubMed and Google Scholar searching for studies published between January 2010 and 2026 using combinations of the terms “extracellular vesicles,” “pregnancy,” and “pollution,” together with related keywords referring to environmental exposures and the maternal–placental–fetal context. To capture studies published before the adoption of the current MISEV-recommended nomenclature, earlier terms commonly used to describe EV subtypes, including “exosomes” and “microvesicles,” were also included in the search strategy. Studies were considered eligible when they investigated EV abundance, characterization, cargo, function, or biological effects in human pregnancy, placental/fetal-membrane models, or experimental pregnancy models, with particular emphasis on environmental exposures. Studies focusing exclusively on non-pregnancy EV biology or without a direct or clearly defined relevance to maternal–fetal or placental biology were not considered for the evidence tables. Eligibility was restricted to studies published in English.

## 2. EV Biogenesis and Classification

EVs are particles enclosed by a phospholipid bilayer. They were previously thought to be cellular debris, but in the last decade, they have gained significant importance as carriers of bioactive molecules. EVs differ in size, biogenesis, molecular composition, and biological function. This heterogeneity reflects their diverse cellular origins and release mechanisms, resulting in distinct EV subpopulations with potentially different capacities to mediate intercellular communication. Although EV nomenclature continues to evolve, the International Society for Extracellular Vesicles (ISEV), through its MISEV (Minimal Information for Studies of Extracellular Vesicles) guidelines (MISEV2018 and MISEV2023), recommends describing EV populations primarily according to their experimentally defined physical, biochemical, and biological properties rather than relying solely on size or a single molecular marker. Accordingly, the broad term “extracellular vesicle” is generally preferred, complemented by additional descriptors reflecting specific experimentally determined characteristics [18,19]. Terms such as “exosome” and “microvesicle” should therefore be reserved for EV populations whose endosomal or plasma-membrane origin, respectively, has been experimentally demonstrated. In the absence of evidence, operational descriptors such as “small EVs” (sEVs) or “large EVs” (lEVs), accompanied by the relevant size range and isolation procedure, are preferable. Nevertheless, characterizing EV populations and investigating the molecular mechanisms governing their biogenesis and release have provided an important framework for understanding their heterogeneity and biological functions. From this perspective, the biogenesis pathways historically associated with endosomal-derived EVs and plasma-membrane-derived EVs are discussed below, while recognizing that isolation procedures and commonly used EV markers alone are generally insufficient to establish these origins.

EVs historically referred to as “exosomes” originate from an endosomal pathway involving the formation of multivesicular bodies (MVBs), which contain intraluminal vesicles (ILVs) that can be released into the extracellular space following the fusion of the MVB with the plasma membrane. However, “exosomes” should be used only when endosomal origin has been demonstrated. The molecular composition of sEV preparations reflects both their cell of origin and the mechanisms involved in their biogenesis. Indeed, sEV preparations may contain membrane proteins such as the tetraspanins CD9, CD63, and CD81, as well as proteins associated with endosomal trafficking and vesicle formation, including tumor susceptibility gene 101 (TSG101) and ALG-2-interacting protein X (ALIX). However, the presence of these proteins does not by itself establish an endosomal origin or justify their designation as “exosomes” since these components can also be detected in other EV populations and therefore lack sufficient specificity to define their biogenetic origin. EVs historically referred to as “microvesicles,” “ectosomes,” or “microparticles” are generally described as originating from outward budding and subsequent fission of the plasma membrane. In this review, as noted above, “microvesicles” should be used only when plasma-membrane origin has been demonstrated. Operationally, populations enriched with larger particles may instead be described as lEVs, with the size range and isolation procedure explicitly reported. lEV preparations can display a variety of cell-type-dependent surface proteins, including integrins, selectins, and CD40 ligands, as well as other membrane-associated proteins such as ARF-6 and cytoskeletal proteins [20].

Finally, apoptotic bodies are generated during the canonical apoptotic cell death pathway and form a distinct class of EVs associated with apoptotic cell fragmentation. They are generally larger than many other EV populations and may contain cellular components derived from the apoptotic cell, including nuclear fragments, cytoplasmic organelles, and other cytoplasmic material, which can subsequently be transferred to or recognized by neighboring or phagocytic cells [21].

**Figure 1:**
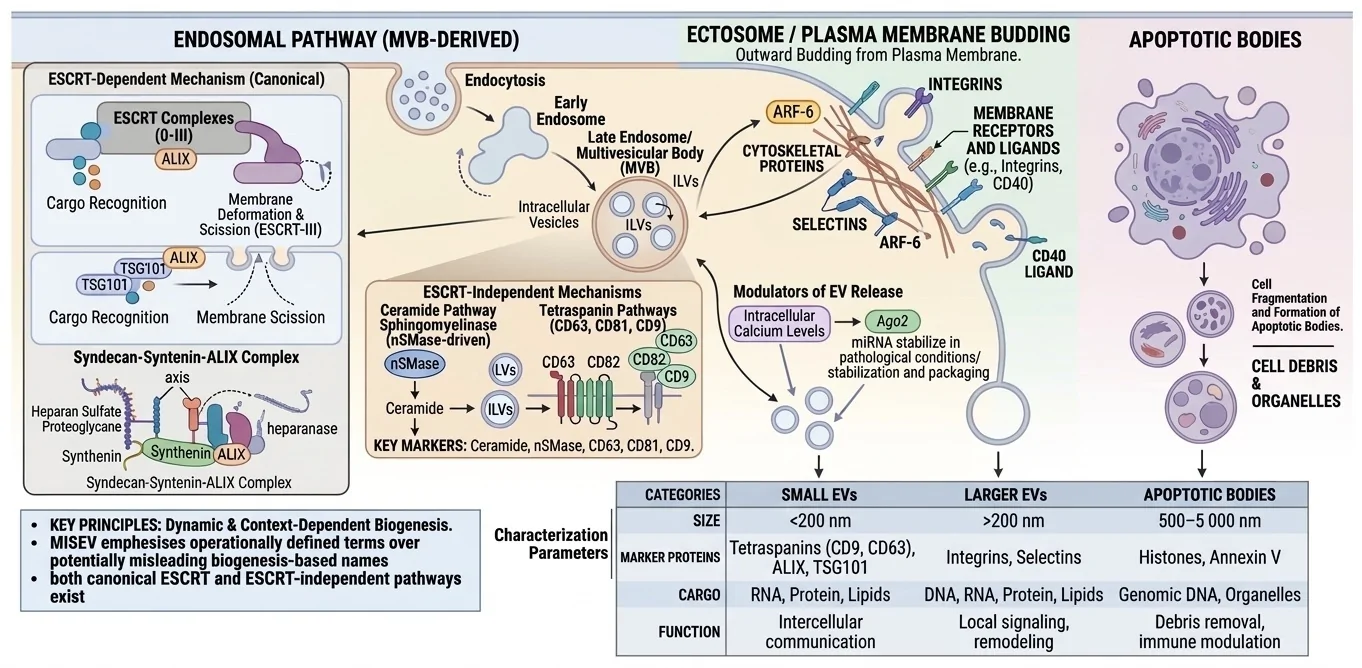
Extracellular vesicle biogenesis and classification. Schematic overview of EV biogenesis through endosomal pathways, outward budding of the plasma membrane, and apoptotic cell death. The molecular mechanisms underlying ESCRT-dependent and ESCRT-independent pathways are summarized on the left side of the figure. EV nomenclature and characterization parameters are presented at the bottom of the figure. This figure was generated using Google Gemini AI.

EV biogenesis is a dynamic and highly regulated process that varies depending on the cell type and its physiological or pathological state [22]. This complex process involves several regulatory pathways and mechanisms that influence both the composition of the cargo and the functions of these vesicles in intercellular communication. Endosomes, which are vesicular structures originating from the plasma membrane through endocytic processes, are a major source of EVs released through the endosomal pathway. Their biogenesis is regulated by several molecular machineries, among which the Endosomal Sorting Complex required for transport (ESCRT) is one of the best-characterized pathways. The ESCRT machinery comprises several protein complexes, including ESCRT-0, ESCRT-I, ESCRT-II, and ESCRT-III. Specifically, ESCRT-0, ESCRT-I, and ESCRT-II are primarily involved in cargo recognition and sorting, whereas ESCRT-III contributes to membrane deformation and the final scission of the budding membrane [20]. EV biogenesis is further regulated by complex interactions between membrane proteins, lipids, and cytoskeletal components. Among the molecular factors involved in EV cargo regulation, Argonaute 2 (Ago2) has been implicated in the stabilization and packaging of miRNAs into EVs, thereby protecting them from extracellular degradation [23]. In addition, enzymes such as phospholipase D2 (PLD2), as well as intracellular calcium levels, can modulate EV biogenesis and release in a context-dependent manner. In particular, alterations in intracellular calcium concentrations have been associated with enhanced EV release under pathological conditions, including cancer [20,24]. The modulation of EV release can implicate an important regulatory axis that involves syndecans, heparan sulfate proteoglycans that interact with tetraspanins [25]. The activation of this pathway frequently requires heparanase and the syndecan–syntenin–ALIX complex, which influences the selection of cargo [26]. Specifically, syntenin binds to syndecan, while ALIX recruits ESCRT-I and -III complexes to promote membrane budding and scission [27]. Although ESCRT-dependent mechanisms are among the best-characterized pathways involved in EV biogenesis, several ESCRT-independent mechanisms have also been described. A lipid-driven biogenesis process is controlled by the enzyme neutral sphingomyelinase (nSMase), which catalyzes the hydrolysis of sphingomyelin to ceramides, thereby inducing the formation of intraluminal vesicles (ILVs) [28,29,30]. Although the complete mechanism is still unclear, the role of ceramides appears to be strongly cell-type-dependent [30]. Another proposed pathway involves tetraspanin-mediated mechanisms in which proteins such as CD63, CD82, and CD9 have been shown to influence EV assembly and secretion in specific cell lines, including melanoma cells and HEK293 cells [27]. These molecular pathways provide evidence for mechanisms that can contribute to EV biogenesis, but their presence or detection in an isolated EV preparation should not, by itself, be interpreted as proof of a specific EV subtype or biogenetic origin. Collectively, these observations indicate that EV biogenesis is not a constitutive housekeeping process but a highly regulated cellular program that determines both the quantity and molecular composition of vesicles released into the extracellular space. Figure 1 provides a schematic overview of EV biogenesis pathways, highlighting the underlying molecular mechanisms, EV nomenclature and characterization parameters described in this section.

## 3. EV-Mediated Maternal–Fetal Communication Throughout Healthy Pregnancy

Pregnancy is a unique and highly dynamic biological state in which maternal and fetal systems undergo profound molecular, metabolic, vascular, and immunological adaptations to support fetal development. Central to this process is the placenta, a transient and highly specialized organ that forms the principal interface between mother and fetus. Beyond its classical roles in nutrient and gas exchange, endocrine regulation, and waste removal, the placenta actively releases physiological signals, integrating maternal and fetal signals to maintain pregnancy homeostasis.

From this perspective, successful human pregnancy can be regarded as a communication-driven biological process in which two genetically distinct organisms continuously exchange molecular information. This dynamic dialogue coordinates implantation, placental development, vascular remodeling, maternal immune tolerance, and fetal growth.

Communication among the maternal, placental, and fetal compartments relies on multiple complementary signaling systems. Endocrine networks orchestrate systemic physiological adaptations, whereas cytokines, chemokines, growth factors, metabolites, cell-free nucleic acids, and receptor-mediated interactions regulate local cellular responses. Direct cell-to-cell contact and selective transport across the placenta provide additional levels of regulation.

Consistent with this, EVs at the maternal–fetal interface are enriched in surface molecules that promote selective delivery to recipient cells, thereby directing the targeted delivery of molecular information [31]. Moreover, these molecules can engage receptors on recipient cells and initiate signaling cascades independently of cargo transfer [32]. At the maternal–fetal interface, experimental observations are broadly consistent with this conceptual framework. Syncytiotrophoblast-derived EVs display an integrated repertoire of immunomodulatory and angiogenic surface molecules, which are described in detail below [33,34,35].

In this view, syncytiotrophoblast-derived EVs may contribute to the dynamic adaptation of maternal tissues throughout gestation. Their changing abundance and molecular cargo, shaped by gestational age and placental condition, suggest that EV-mediated communication may provide a flexible mechanism for adjusting maternal cellular responses to the evolving requirements of pregnancy [36]. Although direct evidence for a coordinating role in vivo remains limited, accumulating experimental findings indicate that gestational changes in EV characteristics are associated with distinct functional responses in recipient maternal cells [37].

The earliest stages of pregnancy require the rapid establishment of a maternal environment that can simultaneously permit embryo implantation, support trophoblast invasion, and initiate extensive vascular remodeling. Evidence from in vitro and ex vivo models consistently indicates that endometrial-derived EVs are internalized by trophoblast cells and preimplantation embryos, where they simultaneously influence adhesion, migration, developmental competence, and tissue remodeling [38,39]. These findings support the idea that the maternal endometrium conveys coordinated molecular programs through EVs to complement classical endocrine and paracrine signaling during implantation [40]. Conversely, trophoblast- and trophectoderm-derived EVs have been shown to modulate maternal endothelial and decidual stromal cells by stimulating endothelial proliferation, migration, and tube formation as well as by delivering angiogenic mediators and regulatory miRNAs [41]. In parallel with these structural changes, maternal immune tolerance must be rapidly established at the implantation site to prevent immune recognition of the semi-allogeneic conceptus. In vitro and ex vivo co-culture studies have shown that trophoblast-derived EVs expressing HLA-G engage inhibitory receptors on decidual natural killer cells (NK cells), reducing their cytotoxic activity while preserving their capacity to support spiral artery remodeling [42]. This localized NK-cell-directed mechanism is the earliest layer of EV-mediated immune adaptation; how it is subsequently extended and maintained across gestation is addressed below [43,44].

As pregnancy progresses, maternal immune tolerance must be actively maintained in the face of expanding placental tissue and a corresponding increase in fetal antigen exposure. Beyond the NK-cell-directed mechanism established at implantation, trophoblast-derived EVs engage additional immune cell populations through complementary pathways as gestation advances [45,46]. Ex vivo experiments with functional co-culture assays using first-trimester and term placental explants have shown that trophoblast-derived EVs carry membrane-bound Fas ligand (FasL) and TNF-related apoptosis-inducing ligand (TRAIL), which retain biological activity and induce the apoptosis of activated maternal T lymphocytes [35]. In parallel, EVs released by placental explants across gestation express the immune checkpoint molecules PD-L1 and B7-H3, providing a receptor-mediated brake on T-cell activation that persists from early to late pregnancy [33]. Beyond these direct receptor–ligand interactions, trophoblast-derived EVs are internalized by decidual macrophages in co-culture where transferred miRNAs and other bioactive cargo modulate macrophage polarization and cytokine output, indicating that EV-mediated communication contributes an innate-immune layer to the tolerogenic environment that complements the T-cell- and NK-cell-directed mechanisms described above. Collectively, these findings indicate that placental-derived EVs coordinate maternal immune adaptation through mechanisms that are established at implantation and subsequently reinforced and diversified as gestation proceeds, acting in parallel on NK cells, T lymphocytes, and macrophages rather than through a single pathway operating uniformly throughout pregnancy [47].

Following implantation, placental development proceeds with the coordinated expansion of maternal and fetal vascular networks together with the progressive differentiation of trophoblast lineages, processes that establish the structural and functional architecture of the mature placenta. Increasing experimental evidence indicates that EVs actively participate in this developmental phase by coordinating communication between trophoblasts, endothelial cells, and decidual stromal cells [48,49]. Experimental evidence from primary trophoblast cultures and first-trimester placental explants demonstrate that trophoblast-derived EVs directly modulate endothelial cell function [49]. Upon internalization, these vesicles enhance endothelial proliferation, migration, and capillary-like network formation, supporting their involvement in the vascular remodeling that accompanies early placental development [48,49]. Beyond their ability to promote endothelial activation, trophoblast-derived EVs transport a complex molecular cargo comprising angiogenesis-related proteins, regulatory miRNAs, lipids, and other bioactive molecules [14,50,51]. Proteomic, lipidomic, and transcriptomic analyses have demonstrated that placental-derived EVs contain multiple members of the chromosome 19 miRNA cluster (C19MC) together with proteins involved in vascular regulation [52,53]. Under pathological conditions such as preeclampsia, syncytiotrophoblast-derived EVs have also been shown to carry anti-angiogenic mediators, including soluble fms-like tyrosine kinase-1 (sFlt-1). Collectively, these findings suggest that placental EVs integrate both stimulatory and inhibitory angiogenic signals, thereby contributing to the dynamic regulation of vascular remodeling throughout gestation [53,54].

**Figure 2:**
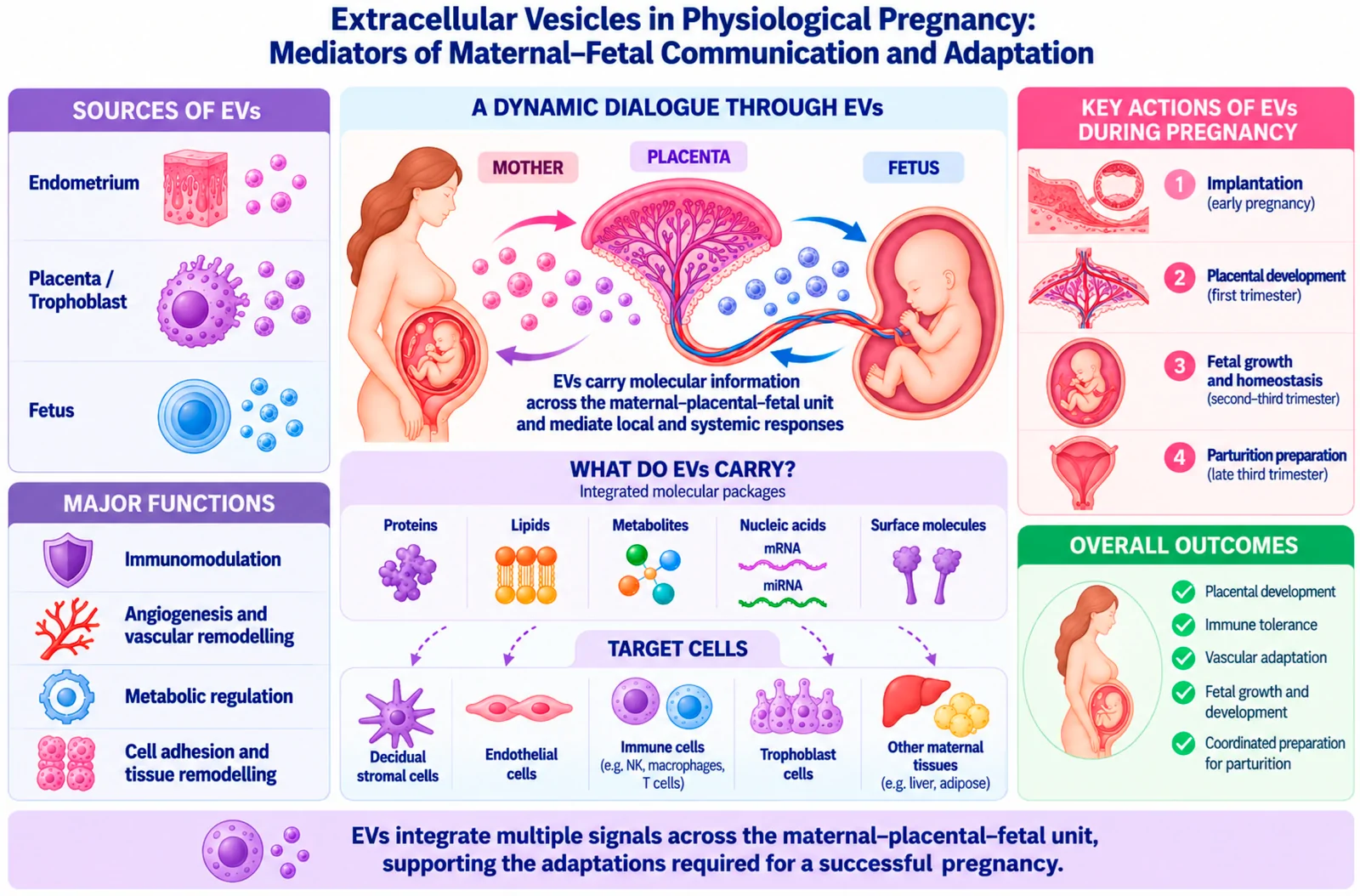
Extracellular vesicles in pregnancy: physiological roles. Schematic representation of the proposed and experimentally supported roles of EVs in maternal–fetal communication throughout pregnancy. The depicted functions are supported by varying levels of experimental evidence. Image generated by ChatGPT (OpenAI, GPT-6, 2026).

The longitudinal sampling of maternal plasma has shown that the concentration of placenta-derived EVs rises progressively across gestation, with a further, steeper increase in the weeks immediately preceding delivery [50,55]. This increase is not limited to number of particles: in a mouse model, maternal plasma EVs isolated on sequential gestational days carried a progressively higher burden of pro-inflammatory mediators between gestation day 5 and day 19, with the shift becoming most pronounced in the final days before parturition. Functionally, EVs isolated from late-gestation (E18) mice and injected into mid-gestation (E15) recipients localized to maternal reproductive tissues and fetal compartments and were sufficient to induce preterm birth-like effects, providing direct experimental evidence that late-gestation EVs can actively drive, rather than merely accompany, the transition to labor [56]. Figure 2 summarizes the current understanding of EV-mediated communication during physiological pregnancy, highlighting the varying levels of evidence supporting their proposed functions. Table 1 provides an overview of the experimental evidence supporting these roles throughout gestation.

## 4. Environmental Exposures and EV Alterations During Pregnancy: Human and Experimental Evidence

As previously described, the molecular cargo of placental EVs contributes to the complex cellular interactions underlying placental function and adaptation during pregnancy. Because these functions depend on the tightly regulated biogenesis, release, and molecular composition of EVs, environmental factors capable of perturbing these processes may affect placental development and maternal–fetal communication during pregnancy. Indeed, the placenta is not merely a passive barrier to environmental stressors; it can modulate their transfer and biological effects while generating molecular signals that influence both maternal and fetal tissues. This bidirectional communication is particularly relevant in the context of environmental exposures as pollutants and other stressors may alter placental function and, in turn, reshape signaling across the maternal–placental–fetal unit [58]. Understanding the pregnancy exposome therefore requires considering not only molecular damage or adaptive responses within individual cells, but also the intercellular communication networks through which exposure-related signals may be transmitted across the maternal–placental–fetal unit.

Interpreting associations between environmental exposures and EV abundance or cargo during pregnancy requires the careful consideration of multiple potential sources of confounding and measurement error. EV profiles vary according to gestational age, maternal body mass index, metabolic status, smoking, medication use, diet, infection or inflammation, pregnancy complications, fetal sex, socioeconomic factors, and pre-analytical variables such as sample collection, processing, and storage. These factors may independently influence circulating or placenta-enriched EV populations and thereby bias estimated exposure–EV associations if not adequately accounted for. Despite the rapidly expanding experimental literature, epidemiological evidence directly linking prenatal environmental exposures to alterations in EV biology during human pregnancy remains limited. Most available studies have relied on in vitro trophoblast models or ex vivo placental explants exposed to particulate matter, cigarette smoke extract, or endocrine-disrupting chemicals (EDCs), whereas relatively few prospective birth cohorts have isolated and characterized EVs from maternal or placental samples in relation to objectively measured environmental exposures. Moreover, human epidemiological studies conducted to date have focused predominantly on EV-associated miRNAs, while other biologically relevant cargo components, including proteins, lipids, and metabolites, remain comparatively underexplored in pregnancy cohorts. Together, these limitations highlight the need for well-characterized longitudinal studies integrating objective exposure assessment with comprehensive EV profiling to clarify how environmental factors may influence EV-associated molecular profiles and, where functionally demonstrated, EV-mediated maternal–fetal communication during pregnancy.

In this context, we critically review the most recent literature on cohort studies, as well as in vitro and ex vivo experimental evidence, to provide an integrated overview of how environmental exposures may influence EV biology during pregnancy.

### 4.1. Environmental Exposures and EV Alterations in Prospective Pregnancy Cohorts

Ambient air pollution is currently the environmental exposure with the strongest epidemiological evidence linking prenatal exposure to alterations in EV biology and function. Several prospective pregnancy cohorts have demonstrated that maternal exposure to particulate matter modifies both circulating and placental-derived EV cargoes, particularly EV-associated miRNAs involved in placental development and vascular homeostasis.

One of the most comprehensive investigations was conducted on the MADRES (Maternal and Developmental Risks from Environmental and Social Stressors) cohort in which Foley and coworkers, relying on residential air pollution models, profiled plasma EV-associated miRNAs in 461 pregnant women and evaluated their relationship with residential exposure estimates for PM_2.5_, PM_10_, NO_2_, O_3_, and NOx [59]. Associations were observed primarily for PM_2.5_ and PM_10_ exposure averaged over the eight weeks preceding late-pregnancy sampling, whereas shorter exposure windows and exposures assessed during early pregnancy were not consistently associated with EV-miRNA alterations. This temporal pattern is important because it suggests that EV-associated cargoes may integrate exposure over a biologically relevant window rather than simply reflect short-term fluctuations in pollutant concentrations. Moreover, several of the altered EV-miRNAs are expressed in placental tissue and participate in pathways involved in trophoblast invasion, vascular remodeling, and preeclampsia, providing biological plausibility for an association between maternal air pollution exposure, EV remodeling, and placental dysfunction.

Further support for this concept comes from the independent Shanghai Maternal–Child Pairs Cohort in which personal exposure to PM_2.5_ during pregnancy was associated with alterations in the miRNA profiles of placental sEVs collected at delivery. In particular, personal exposure reduced certain forms of exposure misclassification, although residual confounding by socioeconomic factors and correlated pollutants remains possible [60].

Importantly, these exposure-related EV-miRNA signatures were subsequently associated with neurodevelopmental delays during early childhood, providing one of the few longitudinal studies linking environmental exposure, placental EV alterations, and offspring health within the same cohort. This suggests that EVs may be one of the molecular interfaces through which environmental exposure during pregnancy can become biologically connected to developmental trajectories extending beyond birth, but do not establish EV-mediated causality.

The potential persistence of exposure-related EV signatures beyond pregnancy is further suggested by findings from the MADRES cohort. Holzhausen and coworkers reported differential expression of EV-associated miRNAs in human milk collected one month postpartum in relation to prenatal PM_10_ exposure [61]. These findings extend the concept of EVs as exposure-responsive biological compartments from pregnancy into the postnatal period and raise the possibility that environmentally induced changes in EV cargo may also influence mother–infant communication after delivery. However, because these EVs were isolated from breast milk rather than directly from placental tissue, their relationship with placental alterations remains uncertain.

Moreover, virtually no prospective cohort has simultaneously integrated quantitative exposure assessment, EV release and characterization, molecular cargo profiling, and longitudinal pregnancy or child health outcomes. Equally noteworthy is the absence of cohort studies investigating tobacco smoke, despite cigarette smoke being among the most extensively studied environmental stressors in experimental studies of placental and uterine EV biology in vitro.

In the MADRES cohort, Howe and coworkers investigated the association between maternal urinary concentrations of multiple metals and circulating EV-associated miRNAs, identifying significant associations for several toxic elements, particularly barium, mercury, and thallium. Pathway analyses indicated the enrichment of biological processes involved in placental development, angiogenesis, and trophoblast differentiation, while mixture modeling demonstrated coordinated effects of combined metal exposure on EV-associated miRNA regulation [62].

Importantly, all cohort studies were conducted using circulating EVs, which represent a heterogeneous population originating from multiple maternal tissues and cell types. Consequently, exposure-associated changes in EV cargo should be interpreted with caution, as they cannot be unequivocally attributed to placenta-derived EVs or considered direct evidence of placental responses to environmental exposures.

Similarly, Zhong and coworkers investigated EV preparations enriched for placenta-associated vesicles from the maternal plasma of women with uncomplicated dichorionic twin pregnancies and related their miRNA cargo to maternal urinary concentrations of phthalate metabolites. Specifically, increased exposure to monobenzyl phthalate and methyl paraben was associated with a higher expression of miR-518e, whereas parabens, triclosan, and dichlorophenols were associated with the reduced expression of miR-543, suggesting that EDCs other than air pollutants can modify the molecular cargo of placenta-enriched EV populations in vivo [63].

Additional support for the broader concept of exposure-related placental miRNA modulation derives from the National Children’s Study Vanguard birth cohort in which placental miRNA expression was associated with prenatal exposure to several toxic metals, including cadmium, mercury, and lead, together with persistent organic pollutants (POPs) including polybrominated diphenyl ethers and polychlorinated biphenyls [64]. Because these analyses were performed on whole placental tissue rather than EV-enriched fractions, they cannot establish whether the observed miRNA alterations are specifically mediated by EVs. Nevertheless, they support the broader concept that environmental exposures can modify placental regulatory pathways that overlap with those identified in EV-based studies.

A similar distinction is necessary when considering POPs and EDCs. PCBs, PBDEs, dioxins, bisphenol A, and phthalates can reach the placenta and interfere with pathways involved in endocrine regulation, oxidative stress, inflammation, and epigenetic control [65,66,67]. Recent studies have shown that prenatal exposure to PCBs and PBDEs is associated with impaired fetal development, epigenetic alterations, and the disruption of stress-responsive signaling pathways such as p38 MAPK [17,68,69,70,71,72]. Although direct evidence linking POP exposure to alterations in EV abundance, composition, or function during human pregnancy remains limited, these findings suggest that POP-induced placental molecular alterations may also affect EV-mediated maternal–fetal communication. Although human evidence remains limited, overall, these data support the hypothesis that EDCs and POPs may alter signaling at the maternal–fetal interface, with EVs emerging as potential molecular indicators of pollutant-induced placental stress, although their role as functional mediators remains to be established.

### 4.2. Placental EVs as Mediators of Pollutant-Induced Placental Dysfunction: In Vitro and Ex Vivo Studies

Despite the limited number of cohort studies investigating the effects of environmental exposures on EV-mediated signaling during pregnancy, growing in vitro and ex vivo evidence provides important mechanistic insights into how environmental pollutants, including particulate matter (PM), polybrominated diphenyl ethers (PBDEs), bisphenols, and other brominated compounds, may modulate EV release, abundance, and molecular cargo, as well as processes involved in EV biogenesis.

For instance, an in vitro study performed by Familari and coworkers on first-trimester trophoblast cells (HTR-8/SVneo) demonstrated that these cells readily internalize PM_2.5_ and PM_10_ particles, leading to oxidative and endoplasmic reticulum (ER) stress, inflammation, impaired proliferation, and altered endocrine function. These cellular alterations closely resemble the pathological features of placental disorders such as preeclampsia and fetal growth restriction, suggesting that pollutant-induced mechanistic alterations in trophoblasts may contribute to impaired placental development, a hallmark of such pregnancy complications.

In addition, exposure to PM during early pregnancy versus chronic exposure may result in distinct effects on both the concentration of circulating EVs and their molecular cargo. Indeed, maternal exposure to PM_2.5_ and PM_10_ during the first trimester was associated with a significant increase in circulating EV populations carrying cell-associated markers, including Syncytin-1^+^ EVs, interpreted as trophoblast-associated EVs, CD105^+^ and CD62E^+^ EVs associated with endothelial cells, CD14^+^ EVs associated with monocytes, and CD25^+^ EVs associated with regulatory T cells [73]. In contrast, chronic exposure was characterized by a reduction in total circulating EV concentrations together with extensive remodeling of the EV-associated miRNA cargo. These alterations affected miRNAs involved in the regulation of angiogenesis, inflammation, endothelial homeostasis, and placental development, targeting key signaling pathways including Fms-like tyrosine kinase-1 (FLT1), Endothelin-1 (EDN1), Angiotensinogen (AGT), and Apelin (APLN) [74]. Among the dysregulated miRNAs, miR-221-3p was significantly downregulated, a finding of particular clinical relevance because reduced miR-221-3p expression is a well-established molecular hallmark of preeclampsia.

Functional in vitro studies have demonstrated that physiological levels of miR-221-3p are essential for trophoblast proliferation, migration, and invasion, indicating that PM-induced repression of this miRNA may impair early placentation and contribute mechanistically to the development of hypertensive disorders of pregnancy. Furthermore, Syncytin-1^+^ EVs, used as a marker-enriched population associated with placental/trophoblast origin, were inversely associated with circulating soluble vascular cell adhesion molecule-1 (sVCAM-1), suggesting a potential association between Syncytin-1^+^ EV populations and endothelial activation [74,75]. Although miR-221-3p has established roles in trophoblast biology and its alteration in pollutant-responsive EVs is supported by current evidence, a causal link between this alteration and hypertensive disorders of pregnancy has yet to be demonstrated in vivo. It has also been demonstrated that environmental toxicants can modulate the biological content of placental EVs without affecting the quantity in which they are released. In an ex vivo human placental explant model, Sheller-Miller and co-workers observed that exposure to PBDEs (congeners 47, 100, 153, 209) and bisphenols (TBBPA, TBP, or BPA) for 24 h did not significantly alter EV concentration or morphology, but did profoundly reshape their proteomic cargo [17]. Specifically, the EV-enriched preparations, referred to as “exosomes” by the authors, were enriched in proteins associated with oxidative stress, inflammation, and cellular injury, including high mobility group box 1 (HMGB1), p38 mitogen-activated protein kinase (MAPK14), and glycogen synthase kinase 3β (GSK3β), while pathways involved in cell survival and tissue repair were suppressed. Furthermore, Shepherd and colleagues demonstrated that EVs released by cigarette-smoke-exposed decidual and myometrial cells crossed the maternal–fetal interface and elicited fetal inflammatory responses through the activation of inflammatory signaling pathways in fetal tissues [76].

These findings suggest that placental EVs provide a molecular fingerprint of placental stress and may represent potential minimally invasive biomarkers of environmental exposure or placental responses, although their ability to predict placental dysfunction before the onset of clinical complications remains to be established in real-life scenarios.

Furthermore, mechanistic studies have provided evidence for functional roles of placental EVs that extend beyond their potential use as biomarkers, although direct causal evidence remains limited to specific experimental models. Liu and coworkers demonstrated that prenatal exposure of pregnant C57BL/6J mice to triphenyl phosphate (TPHP), an organophosphate flame retardant, altered both the production and molecular cargo of trophoblast-derived EVs. These vesicles were enriched in PPARγ and were subsequently transferred to fetal hepatocytes where EV-associated PPARγ activated lipogenic pathways, promoted hepatic lipid accumulation, and resulted in persistent hyperlipidemia in offspring. Importantly, the pharmacological inhibition of PPARγ attenuated these metabolic effects, providing direct evidence that, in this model, pollutant-modified placental-derived EV cargoes can contribute to fetal metabolic programming, supporting a functional role for EV-mediated cargo transfer [77].

**Figure 3:**
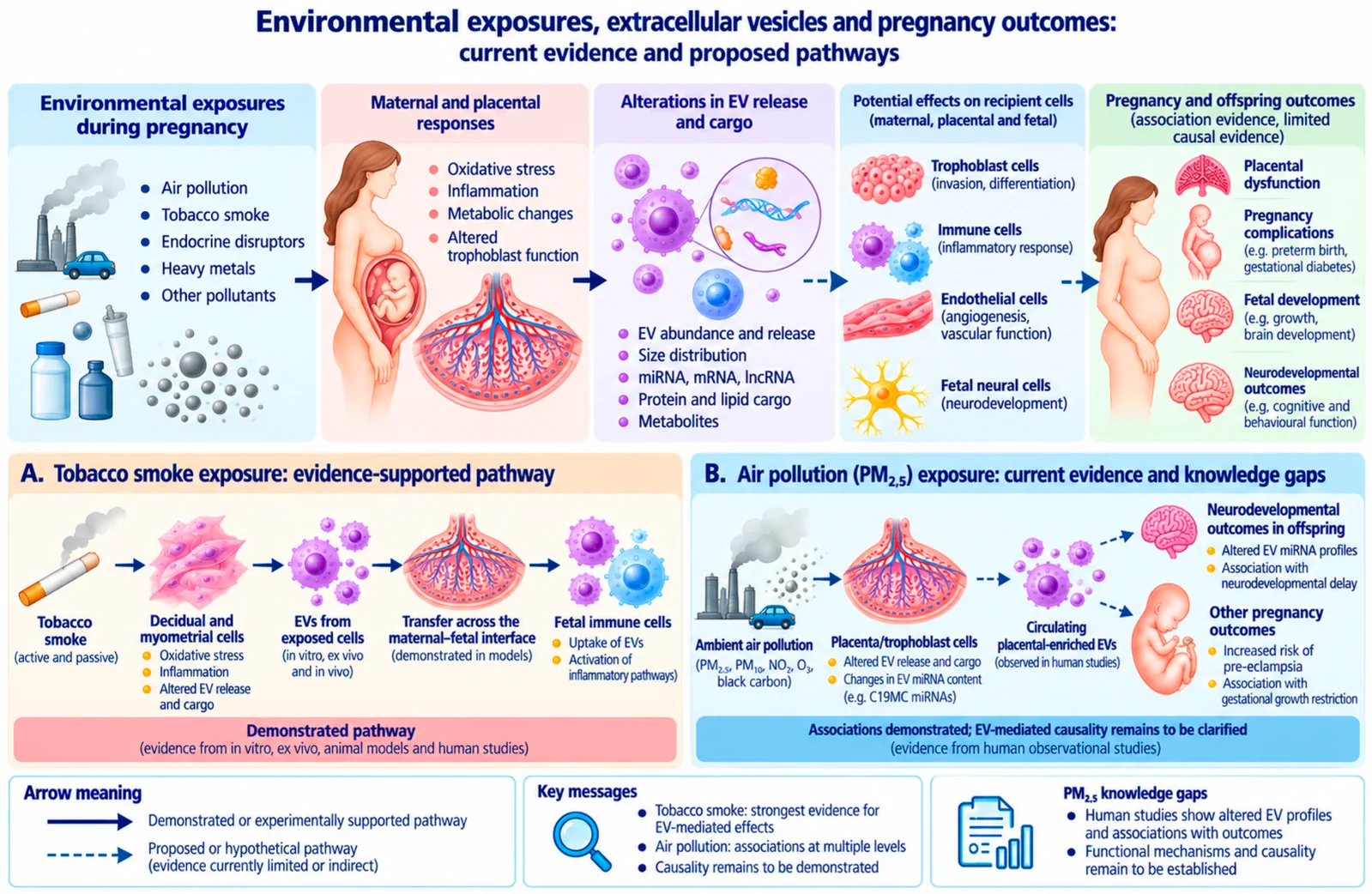
Schematic representation of potential mechanisms by which environmental pollutants alter EV biogenesis, release, and cargo composition at the maternal–placental–fetal interface, and associated adverse pregnancy and offspring outcomes. Image generated by ChatGPT (OpenAI, 2026).

Consistent with this concept, Kahroba and coworkers’ recent review proposes that placental sEVs are a central mechanistic link connecting maternal air pollution exposure to placental dysfunction, fetal epigenetic programming, and increased susceptibility to chronic diseases during childhood and adulthood [78]. Together, these findings support a model in which environmental pollutants induce oxidative stress and inflammatory signaling within maternal tissues and the placenta, resulting in changes in EV release and molecular cargo. Experimental evidence in human placental explants demonstrates that environmental pollutants can substantially modify EV protein cargo, including proteins associated with cellular stress, inflammation, and tissue injury [17]. In contrast, mechanistic studies in non-placental systems indicate that specific pollutants can modulate EV release through distinct cellular pathways, including aryl hydrocarbon receptor (AhR)-dependent mechanisms in response to polycyclic aromatic hydrocarbons and P2X7-dependent signaling in response to particulate matter [79,80]. However, direct evidence that environmental pollutants alter specific molecular pathways governing EV biogenesis in placental cells remains limited. Nevertheless, important challenges remain, including the need for standardized EV separation and characterization protocols, identification of pollutant-specific EV signatures, and prospective longitudinal studies to establish causal relationships between environmental exposures, placental EV alterations, and pregnancy and offspring outcomes. Figure 3 summarizes the current evidence along the proposed exposure–EV–outcome pathway, distinguishing experimentally supported mechanisms from observational associations and from biologically plausible but insufficiently validated links. Environmental exposures have been associated with altered EV abundance and molecular cargo, and selected experimental models support functional EV-mediated effects. However, as the figure shows, several links in the causal sequence from environmental exposure to EV alterations and subsequent placental or offspring outcomes remain to be drawn. Table 2 provides an overview of the litterature evidence linking EV to environmental exposures.

**Table 2:** Critical synthesis of EV evidence strength in term of environmental exposure.

Reference	Species/Model	EV Isolation and Characterization	Placental Origin Demonstrated	Functional Validation	Type of Inference	Main EV-Related Findings	Relevance to EV Environmental Exposure
[17]	Ex vivo: Human placental explant culture	Placental-explant exosomes; NTA/particle characterization & proteomics	Yes (human placental tissue explant)	No (proteomic cargo profiling)	Association * and Mechanistic **	Contaminants altered exosomal protein cargo (HMGB1, MAPK14, GSK3β) without changing particle count or morphology	Demonstrates environmental contaminants reshape placental EVs cargo independently of EVs release rate
[52]	Ex vivo: Primary human trophoblasts & circulating plasma	Trophoblast-derived EVs; miRNA profiling	Yes (primary human trophoblasts/C19MC miRNA profile)	No (descriptive/expression profiling)	Association *	C19MC miRNAs predominated in trophoblast-derived EVs, reflecting parental trophoblast miRNA profile	Supports placental EVs miRNAs as biomarkers of trophoblast status and environmental stress
[61]	Human mother–infant cohort & experimental model	Breast milk-derived EVs; miRNA profiling	No (non-placental: breast milk origin)	In vitro (mechanistic link to oxidative stress regulation)	Association * and Mechanistic **	PM exposure increased breast milk EVs miR-200b-3p and miR-378d; miR-200b-3p regulates oxidative stress	Highlights non-placental EV-mediated maternal–infant communication altered by environmental pollution
[73]	In vitro: First-trimester human trophoblasts (HTR-8/SVneo) In vivo: Mouse model	EVs release, endocytosis, cellular protein profile	Yes (trophoblast cell line release)	In vitro (endocytosis and cellular uptake analysis)	Mechanistic **	Early PM exposure altered protein profile and increased EVs release; chronic exposure reduced release	Early PM exposure perturbs pathways in inflammation, ER stress, cell survival, and transport
[74]	Human prospective cohort (INSIDE cohort)	Maternal plasma EVs (Syncytin-1^+^, CD105^+^/CD62E^+^, CD14^+^, CD25^+^); miRNA profiling	Partial/Phenotypic (syncytin-1^+^ subset identifies trophoblast origin)	No (observational human cohort)	Association *	First-trimester PM exposure increased circulating EVs; chronic exposure reduced total EVs and altered miRNA cargo	Supports the potential use of circulating EV-associated miRNAs as candidate biomarkers of exposure-related vascular and placental responses
[75]	In vitro: Trophoblast cell model	None (cellular study, not EV-focused)	No (direct cellular modulation)	In vitro (cellular proliferation, migration, and invasion assays)	Mechanistic ** (non-EV)	miR-221-3p regulates trophoblast migration and invasion; reduced expression impairs placentation	Indirectly supports the biological relevance of PM-associated EV miRNA changes identified in exposed pregnancies
[76]	In vitro: Primary human uterine myometrial/decidual cells In vivo: Pregnant mice	Stressed uterine cell EVs; NTA, Western blot, in vivo fetal inflammation assays	No (maternal uterine/decidual origin, non-placental trophoblast)	Yes (in vivo transfer triggering fetal inflammatory response)	Causal *** and Mechanistic **	Stressed maternal uterine EVs crossed the maternal–fetal interface to activate inflammatory responses in fetal tissues	Demonstrates maternal stress-induced non-placental EVs cross the interface to mediate maternal–fetal signaling
[77]	In vivo: Pregnant C57BL/6J mice In vitro: Validation assays	Placenta/trophoblast EVs; cargo characterization; functional transfer to fetal hepatocytes	Yes (placenta/trophoblast-derived EVs)	Yes (in vivo and in vitro functional transfer assays)	Causal ***	TPHP altered trophoblast EVs cargo, driving PPARγ-dependent fetal hepatic lipogenesis and hyperlipidemia	Provides direct causal evidence that pollutant-modified placental EVs mediate fetal metabolic programming

Note: Table 2 provides an overview of studies investigating the role of EVs in environmental exposures during pregnancy, summarizing the experimental models, EV isolation and characterization methods, evidence of placental origin, study designs, levels of evidence (associative *, mechanistic ** or causal ***), and key findings. * Associative study: Observational or clinical studies correlating EV levels, markers, or molecular cargo with physiological or pathological conditions, without demonstrating functional effects. ** Mechanistic study: In vitro or ex vivo studies demonstrating functional effects of EVs or their cargo on recipient cells under controlled experimental conditions. *** Causal study: In vivo interventional studies demonstrating that EV manipulation or administration directly contributes to specific biological or pathological outcomes. Abbreviations: C19MC, Chromosome 19 MicroRNA Cluster; TPHP, Triphenyl Phosphate; PPARγ, Peroxisome Proliferator-Activated Receptor gamma; HMGB1, High Mobility Group Box 1; MAPK14, Mitogen-Activated Protein Kinase 14; GSK3β, Glycogen Synthase Kinase 3 beta.

## 5. Placenta-Derived EVs as Biomarkers of Placental Health and Environmental Response

As a key interface between the maternal and fetal environments, the placenta continuously responds to environmental cues, generating molecular signals that reflect its functional state and adaptive responses [81,82,83,84]. Among these, placenta-derived EVs are emerging as promising biomarkers of placental health and environmental response.

Despite its pivotal role, the placenta remains one of the least accessible organs during pregnancy. Except for invasive procedures performed for specific clinical indications, such as chorionic villus sampling, direct assessment of placental tissue is generally limited to delivery. Although advances in ultrasonography and magnetic resonance imaging have substantially improved the in vivo evaluation of placental morphology, vascularization, and, more recently, functional parameters such as perfusion and oxygenation, these techniques provide only indirect or macroscopic information and cannot directly interrogate the molecular and cellular processes underlying placental adaptation and dysfunction. Consequently, there is a critical need for minimally invasive biomarkers capable of providing longitudinal insight into placental biology throughout gestation [85,86]. Among the diverse placental signals released into the maternal circulation, EVs have emerged as particularly promising candidates, as they convey integrated molecular information reflective of the functional state of their tissue of origin [87].

The syncytiotrophoblast is the best-characterized placental source of circulating EVs, though evidence indicates that other trophoblast and fetal-membrane populations may also contribute to the circulating vesicle pool [53]. As this highly secretory epithelium simultaneously governs endocrine signaling, nutrient exchange, and immune regulation at the maternal–fetal interface, it has been proposed that membrane shedding might be an additional route of placental communication with the maternal organism [88]. The first experimental support for this idea came from Knight and colleagues, who detected placental alkaline phosphatase (PLAP)-positive syncytiotrophoblast membrane EVs in the peripheral blood of pregnant women, providing evidence that trophoblast-associated membrane vesicles are released into maternal circulation under physiological conditions [89]. Using ex vivo dual perfusion of human placental lobules, Tannetta and colleagues and, later, Dragovic and colleagues demonstrated the continuous release of morphologically and molecularly distinct EV populations from the perfused placenta. These vesicles were enriched in placental markers such as PLAP and canonical EV proteins, including CD63, CD81, TSG101, and ALIX, and were therefore characterized as syncytiotrophoblast-derived EVs [90,91].

Importantly, the ex vivo perfusion model provides a controlled setting in which the placenta is the source of the recovered vesicles, providing direct experimental evidence that PLAP-positive EVs can be released by placental tissue [92].

Subsequent studies have expanded the range of potential trophoblast-associated EVs, showing that first-trimester extravillous trophoblasts can release HLA-G- and HLA-C-bearing sEVs in cellular models. These studies therefore point to multiple potential cellular sources of EVs within the placental and fetal-membrane compartments [93]. However, to date, the available approaches do not allow circulating maternal EVs to be reliably distinguished by their specific cellular origin. Collectively, these observations support two key principles: (i) distinct placental and fetal-membrane cell populations can release EVs carrying cell-associated molecular features; and (ii) these vesicles are detectable in maternal circulation during pregnancy, making them accessible through peripheral blood sampling. However, the identification of cell-type-associated markers should not by itself be considered definitive evidence of EV biogenesis or cellular origin. Accordingly, circulating EV populations identified through placental or trophoblast-associated markers are more conservatively described as placental- or trophoblast-enriched EV populations when direct evidence of origin is lacking. Nevertheless, these findings provide a biological rationale for investigating placental EVs as circulating surrogates of placental physiology and as minimally invasive biomarkers of placental health.

In this context, EVs offer a unique opportunity to bridge the external and internal dimensions of the pregnancy exposome in a minimally invasive biological readout. Although only partially permeable to different classes of pollutants, the placenta can limit fetal exposure to various xenobiotics and activate adaptive responses to counteract their harmful effects. Accordingly, alterations in the abundance or molecular cargo of placenta-derived EVs have the potential to reveal early placental responses to environmental insults before the onset of clinically detectable dysfunction [13]. Although this remains a promising and rapidly growing area of research, several technical challenges must be addressed and standardized before its full potential can be realized.

Unlike EVs collected from conditioned cell culture media or placental explants, maternal plasma or serum contains a highly heterogeneous mixture of EVs originating from multiple maternal tissues and circulating blood cells, including platelets, leukocytes, erythrocytes, and endothelial cells, whereas trophoblast-derived EVs constitute only a minor fraction of the total circulating vesicle population [94,95]. Given the low abundance of these EVs in maternal circulation, approaches capable of enriching trophoblast-derived EVs within the broader circulating vesicle population are often necessary to investigate placenta-associated signals.

Immunoaffinity-based methods targeting trophoblast-specific markers, most notably PLAP and, in selected applications, HLA-G, are therefore often used to improve analytical specificity and enrich EV populations carrying placenta- or trophoblast-associated markers. However, such approaches are inherently marker-dependent: their performance is influenced by antibody specificity and antigen accessibility, while no single marker is expected to capture the full variety of placental-derived EV subpopulations across gestation [96,97]. Recent evidence further challenges the assumption that PLAP is exclusively indicative of placental origin. Kumar and colleagues reported PLAP expression not only in placental trophoblasts, but also in chorion trophoblast cells and their associated EVs, suggesting that PLAP-positive EVs in maternal blood may therefore represent a broader trophoblast-associated or feto-maternal interface derived population rather than a placenta-specific EV fraction. This raises an important methodological challenge for the interpretation of circulating EVs as marker-based enrichment can increase the specificity for a defined while potentially limiting representativeness and restricting the analysis to selected EV subpopulations. These limitations have also driven efforts to identify surface markers that may enable a more selective characterization of EVs associated with specific placental cell populations. Syncytin-1, for example, has emerged as a promising candidate for identifying syncytiotrophoblast-associated EVs, with expression demonstrated in trophoblast-derived EVs and detectable signals in maternal plasma EVs [97]. However, its specificity for syncytiotrophoblast-derived EVs and its ability to provide a robust in vivo readout of this specific EV population remain to be fully validated.

Alternative isolation workflows, including differential ultracentrifugation, are widely used as initial EV separation strategies, often followed by density gradient separation or size-exclusion chromatography to improve purity. However, these approaches isolate EV-enriched preparations rather than biogenetically defined EV subtypes and may co-isolate lipoproteins, protein aggregates, and other non-vesicular plasma components [98].

Collectively, these methodological considerations underline that EV isolation and enrichment are not merely technical steps, but major determinants of biological interpretation. Differences in isolation protocols directly influence vesicle recovery, purity, and molecular composition, representing one of the principal sources of variability among published studies. Consequently, adherence to standardized isolation and characterization workflows and reporting guidelines has become essential for ensuring reproducibility and facilitating cross-study comparisons.

## 6. Conclusions

The evidence reviewed suggests that EVs may reflect environmental stress during pregnancy and participate in maternal–placental–fetal communication, contributing to physiological processes such as immune adaptation and vascular remodeling. However, human studies remain largely associative, and it is unclear whether exposure-related EV changes are biomarkers of stress, mediators of adverse effects, or both. Although selected experimental studies support functional roles for exposure-modified EVs, their contribution to adverse pregnancy and offspring outcomes remains incompletely established. Overall, these findings partially support the hypothesis that EVs may act as indicators of environmental stress and potential mediators of maternal–placental–fetal communication, while highlighting the need for further evidence to establish causal pathways linking environmental exposures to adverse outcomes.

To clarify their role, future studies must reliably identify placenta-derived EVs in maternal blood using validated markers and standardized isolation methods, in accordance with MISEV2023 recommendations. Longitudinal studies linking environmental exposures and EV profiles with pregnancy and offspring outcomes could establish their value as minimally invasive biomarkers of placental responses. Research should consider different classes of environmental exposures and critical windows of susceptibility, while evaluating whether EV-based signatures provide additional diagnostic or prognostic information beyond established clinical indicators.

Complementary mechanistic studies are needed to determine whether altered EVs contribute to adverse outcomes.

## Figures and Tables

**Table 1:** Roles of EVs in physiological pregnancy.

Reference	Species/Model	EV Isolation and Characterization	Placental Origin Demonstrated	Functional Validation	Type of Inference	Main Findings and Synthesis
[13]	Literature review (placental EVs in maternal circulation)	N/A (review of characterization and liquid biopsy approaches)	N/A (summarizes circulating placental EV literature)	N/A (reviews functional impact of EV cargo transfer)	Synthesis/Review	Highlights circulating placental EVs as essential long-distance signaling entities transporting nucleic acids, proteins, and lipids between fetus and mother.
[33]	Human (in vitro tissue explants: 1st trimester & term placenta)	Differential ultracen-trifugation; WB, Flow Cytometry, ELISA	Yes (explant tissue & trophoblast-derived; HLA-G, FasL, immune markers)	In vitro functional immune assays with primary/activated cells	Mechanistic **	Placental exosomes carry HLA-G and FasL to actively suppress/modulate maternal immune cell activation, contributing to fetal immune privilege.
[34]	In vitro cell-line model (immune-tolerant cell lines)	Standard EV isolation; WB, ELISA, molecular expression profiling	No (cell-line model mimicking immune tolerance; indirect relevance)	Molecular & expression analysis of secretome/EVs	Mechanistic **	EV-mediated secretion of HLA-G maintains continuous immune-tolerant signals, outlines molecular mechanisms relevant to maternal-fetal adaptation.
[35]	Human (in vitro explant-derived exosomes & primary immune cells)	Nanoparticle characterization, WB, Flow Cytometry	Yes (human placenta-derived exosomes; functional FasL/TRAIL detection)	In vitro apoptosis assays using activated immune cells	Mechanistic **	Placental exosomes induce apoptosis in activated maternal T cells via surface FasL and TRAIL, establishing an active immunosuppressive mechanism.
[36]	Human (clinical cohort study; maternal plasma)	Flow Cytometry, microparticle quantification, inflammatory cytokine assays	Yes (syncytiotropho-blast microparticles, STBMs, identified in circulating plasma)	Ex vivo inflammatory priming assays on maternal leukocytes	Association * and Mechanistic **	Circulating STBMs increase during normal pregnancy and surge in pathological states, correlating with systemic maternal vascular inflammatory priming.
[37]	Human (longitudinal clinical cohort; maternal plasma)	Ultracentrifugation/isolation, EM, WB, NTA	Yes (placental-derived exosomes tracked longitudinally in maternal plasma)	In vitro endothelial cell migration assays	Association * and Mechanistic **	Circulating placental exosomes increase progressively over gestation and functionally enhance endothelial migration, supporting maternal vascular adaptation.
[38]	Human/in vitro (primary human endometrial exosomes)	Mass spectrometry proteomics, physical EV characterization	No (maternal origin) (Hormone-regulated endometrial exosomes)	In vitro trophoblast adhesion assays	Mechanistic **	Hormonally controlled endometrial exosomal cargo directly enhances trophoblast adhesion, regulating the receptive window for embryo implantation.
[39]	In vitro cell-line model (trophoblast & endometrial cell co-culture)	EV isolation, RNA sequencing, transcriptomic profiling	In vitro trophoblast (cell-line derived trophoblast EVs)	In vitro endometrial cell uptake & functional transcriptomic remodeling assays	Mechanistic **	Trophoblast EVs transfer RNA cargo to endometrial cells to alter gene expression, mediating embryo-maternal crosstalk during implantation.
[40]	Human/in vitro (human endometrial small EVs & trophectoderm invasion model)	Proteomics, phosphoproteomics, physical EV characterization	No (maternal origin) (Endometrial small EVs acting on trophectoderm)	In vitro trophectoderm invasion and phosphoproteome alteration assays	Mechanistic **	Maternal endometrial sEVs remodel the trophectoderm phosphoproteome, regulating invasion and establishing bidirectional maternal-embryo crosstalk.
[41]	Literature review (angiogenesis and placenta-derived EVs)	N/A (review of isolation, omics, and vascular profiling)	N/A (synthesizes literature on placental EVs)	N/A (compiles reported endothelial & angiogenesis assays)	Synthesis/Review	Summarizes how placental EVs promote physiological vascular remodeling and how alterations in EV signaling correlate with preeclampsia development.
[42]	Human (clinical cohort/tissue profiling of decidual NK cells)	Flow Cytometry, receptor expression, immunophenotyping	No/indirect (focuses on dNK cell microenvironment; non-EV direct assay)	Ex vivo receptor expression & cytokine secretion profiling	Association *	Identifies altered dNK cell receptor expression in impaired vascular remodeling, providing essential immunological context for EV-NK interactions.
[43]	In vitro cell culture (trophoblast & primary decidual NK cells)	EV isolation, WB, Flow Cytometry, metabolic profiling	In vitro trophoblast (HLA-E expressing trophoblast-derived EVs)	In vitro dNK cell metabolic regulation & functional activity assays	Mechanistic **	EV-bound HLA-E from trophoblasts targets decidual NK cells, reprogramming their metabolic activity to promote immunotolerance at the maternal-fetal interface.
[44]	Literature Review (extravillous trophoblast & uNK cell interactions)	N/A (review of EV and non-EV immune pathways)	N/A (synthesizes trophoblast and uNK cell cross-talk)	N/A (summarizes functional invasion & vascular assays)	Synthesis/Review	Frames EV-mediated communication within the broader landscape of extravillous trophoblast and uterine NK cell interaction during uterine vascular remodeling.
[48]	Literature review (placental EV biology in pregnancy)	N/A (review of isolation, omics, and functional approaches)	N/A (integrates physiological and pathological studies)	N/A (synthesizes systemic and localized EV functional data)	Synthesis/Review	Integrates current multi-disciplinary knowledge on placental EV biosynthesis, molecular cargo, and systemic homeostatic roles in pregnancy.
[49]	Literature review (placental EV regulation of trophoblast & endothelium)	N/A (review of EV isolation and molecular assays)	N/A (synthesizes literature on placental EVs)	N/A (compiles endothelial and trophoblast functional studies)	Synthesis/Review	Provides an updated comprehensive synthesis of placental EV roles in regulating trophoblast invasion, endothelial responses, and vascular adaptation.
[50]	In vitro model (extravillous trophoblast cell lines)	Exosome isolation, NTA, Proteomics	In vitro trophoblast (extravillous trophoblast line-derived exosomes)	In vitro vascular smooth muscle cell (VSMC) migration assays	Mechanistic **	Extravillous trophoblast exosomes directly stimulate vascular smooth muscle cell migration, demonstrating EV involvement in spiral artery remodeling.
[51]	Literature review (immune tolerance in implantation)	N/A (review of embryonic & decidual EV literature)	N/A (synthesizes embryonic and maternal EV sources)	N/A (compiles immunological and tolerance functional assays)	Synthesis/Review	Emphasizes the bidirectional EV-mediated dialogue between embryonic and maternal decidual stromal cells necessary for establishing immune tolerance.
[55]	Human (longitudinal clinical cohort; maternal plasma)	Exosome isolation, NTA, placental marker profiling (PLAP)	Yes (placenta-specific marker PLAP confirmed on circulating exosomes)	Longitudinal biomarker quantification across first trimester	Association *	Establishes that circulating placenta-derived exosomes continuously increase across early pregnancy, validating them as dynamic biomarkers of physiological adaptation.
[56]	Mouse model (in vivo pregnant mice)	EV isolation, molecular characterization, structural analysis	Yes (mouse placental & maternal exosomes)	In vivo administration of engineered/pathological EVs in pregnant mice	Causal ***	Directly demonstrates that systemic administration of inflammatory or aberrant exosomes causes preterm birth outcomes in vivo, providing causal evidence of pathophysiology.
[57]	Human (clinical case-control cohort; normal vs. preeclampsia)	Exosome isolation, ELISA, Western blot, quantitative profiling	Yes (placental exosomes isolated from maternal circulation)	Comparative quantification and correlation with clinical disease state	Association *	Demonstrates significant differences in concentration and cargo of circulating placental exosomes between preeclamptic and normotensive pregnancies.

Note: Table 1 summarizes experimental models, EV isolation and characterization methods, evidence of placental origin, study design, level of evidence (associative *, mechanistic ** or causal ***), and principal findings of the studies. The table illustrates the contribution of EVs to physiological pregnancy adaptations and pregnancy-related complications. * Associative study: Observational or clinical studies correlating EV levels, markers, or molecular cargo with physiological or pathological conditions, without demonstrating functional effects. ** Mechanistic study: In vitro or ex vivo studies demonstrating functional effects of EVs or their cargo on recipient cells under controlled experimental conditions. *** Causal study: In vivo interventional studies demonstrating that EV manipulation or administration directly contributes to specific biological or pathological outcomes. Abbreviations: HLA-G, Human Leukocyte Antigen G; FasL, Fas Ligand; TRAIL, TNF-Related Apoptosis-Inducing Ligand; STBMs, Syncytiotrophoblast Microparticles; HLA-E, Human Leukocyte Antigen E; VSM, Vascular Smooth Muscle cells.

## Data Availability

No new data were created or analyzed in this study. Data availability is not applicable to this article.

## Abbreviations

The following abbreviations are used in this manuscript:

AGT	Angiotensinogen
Ago2	Argonaute 2
ALIX	ALG-2-Interacting protein X
APLN	Apelin
BPA	Bisphenol A
C19MC	Chromosome 19 MicroRNA Cluster
EDCs	Endocrine-Disrupting Chemicals
EDN1	Endothelin-1
ESCRT	Endosomal Sorting Complex Required for Transport
EVs	Extracellular Vesicles
FasL	Fas Ligand
FLT1	Fms-Like Tyrosine kinase-1
GSK3β	Glycogen Synthase Kinase 3 beta
HEK293	Human Embryonic Kidney 293
HLA-C	Human Leukocyte Antigen C
HLA-G	Human Leukocyte Antigen G
HMGB1	High Mobility Group Box 1
ILVs	Intraluminal Vesicles
ISEV	International Society for Extracellular Vesicles
MADRES	Maternal and Developmental Risks from Environmental and Social Stressors
MAPK	Mitogen-Activated Protein Kinase
MiRNA	MicroRNA
MISEV	Minimal Information for Studies of Extracellular Vesicles
MRI	Magnetic Resonance Imaging
MVBs	Multivesicular Bodies
NCDs	Non-Communicable Diseases
NK cells	Natural Killer cells
NO_2_	Nitrogen Dioxide
NOx	Nitrogen Oxides
nSMase	Neutral Sphingomyelinase
O_3_	Ozone
PBDEs	Polybrominated Diphenyl Ethers
PCBs	Polychlorinated Biphenyls
PDL1	Programmed Death-Ligand 1
PLAP	Placental Alkaline Phosphatase
PLD2	Phospholipase D2
PM_2.5_, PM_10_	Particulate Matter
POPs	Persistent Organic Pollutants
PPARγ	Peroxisome Proliferator-Activated Receptor gamma
sFlt-1	Soluble Fms-like tyrosine kinase-1
sVCAM-1	Soluble Vascular Cell Adhesion Molecule-1
STBMs	Syncytiotrophoblast microparticles
TBBPA	Tetrabromobisphenol A
TBP	2,4,6-Tribromophenol
TPHP	Triphenyl Phosphate
TRAIL	TNF-Related Apoptosis-Inducing Ligand
TSG101	Tumor Susceptibility Gene 101
VSM	Vascular Smooth Muscle cells
